# Recent Advances in Characterization of Lignin Polymer by Solution-State Nuclear Magnetic Resonance (NMR) Methodology

**DOI:** 10.3390/ma6010359

**Published:** 2013-01-23

**Authors:** Jia-Long Wen, Shao-Long Sun, Bai-Liang Xue, Run-Cang Sun

**Affiliations:** 1 Beijing key laboratory of lignocellulosic chemistry, Beijing Forestry University, Beijing 100000, China; E-Mails: wenjialonghello@126.com (J.-L.W.); sunshaolong328@126.com (S.-L.S.); xuebailiang67@163.com (B.-L.X.); 2 State Key Laboratory of Pulp and Paper Engineering, South China University of Technology, Guangzhou 510000, China

**Keywords:** lignin, solution-state NMR, quantitative heteronuclear-single-quantum-coherence spectra (HSQC), *in situ* characterization

## Abstract

The demand for efficient utilization of biomass induces a detailed analysis of the fundamental chemical structures of biomass, especially the complex structures of lignin polymers, which have long been recognized for their negative impact on biorefinery. Traditionally, it has been attempted to reveal the complicated and heterogeneous structure of lignin by a series of chemical analyses, such as thioacidolysis (TA), nitrobenzene oxidation (NBO), and derivatization followed by reductive cleavage (DFRC). Recent advances in nuclear magnetic resonance (NMR) technology undoubtedly have made solution-state NMR become the most widely used technique in structural characterization of lignin due to its versatility in illustrating structural features and structural transformations of lignin polymers. As one of the most promising diagnostic tools, NMR provides unambiguous evidence for specific structures as well as quantitative structural information. The recent advances in two-dimensional solution-state NMR techniques for structural analysis of lignin in isolated and whole cell wall states (*in* *situ*), as well as their applications are reviewed.

## 1. Introduction

Plant lignin currently attracts widespread attention as a feedstock due to its renewability and large abundance. Despite its widespread availability, industrial application of lignin is rather limited [1,2]. Understanding the specific structure, types, sources, reactivity, and preparation methods of lignin is of vital importance for biorefinery. The effective utilization of lignin for a range of natural and industrial purposes is largely dependent on our knowledge of lignin. However, the inherent complexity and heterogeneity of lignin, which hinders the development of an efficient and economical conversion technology of lignocellulosic materials, has not yet been elucidated. 

**Figure 1:**
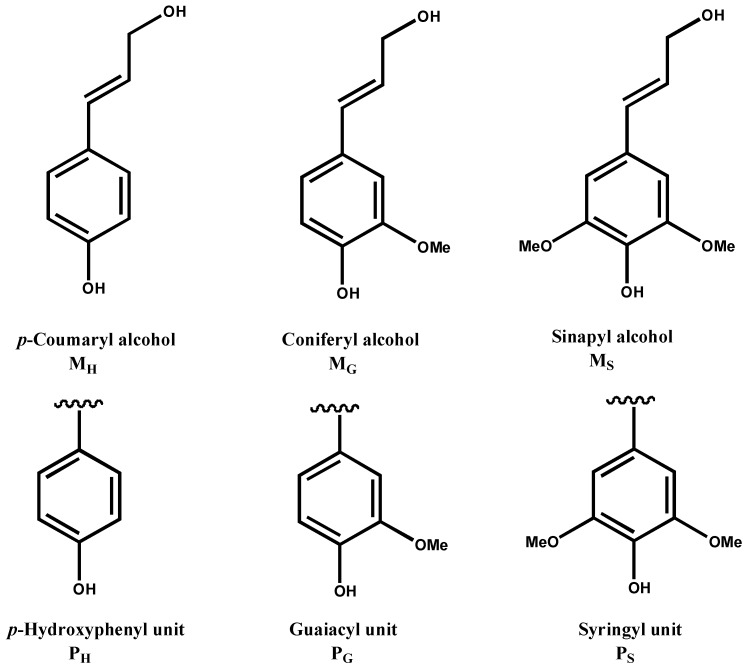
Lignin phenylpropanoid units.

Lignin is located in the plant cell wall together with cellulose and hemicelluloses. It acts as reinforcement for the lignocellulosic matrix and provides rigidity, water-impermeability, and resistance against microbial attack. Its amount in lignified plants ranges from 15% to 36% by mass [3]. It is well accepted that lignin is a phenolic polymer derived primarily from three hydroxycinnamyl alcohols or monolignols, namely, *p*-coumaryl alcohol (MH), coniferyl alcohol (MG), and sinapyl alcohol (MS) (Figure 1). By free radical generation, these monomers assemble into an intricate racemic macromolecule via combinatorial free radical coupling, giving rise to *p*-hydroxyphenyl (H), guaiacyl (G) and syringyl (S) subunits in the polymer [4,5]. Further combinations and crosslinking of these monolignols results in the complex structures of lignin, and the monolignols are incorporated into lignins with species, tissue and developmental specificity. 

Structure analysis of lignin is an important issue in the wood and pulping chemistry. For decades, lignin chemists have devoted their efforts to analyzing lignin polymers. Traditionally, the “original lignin” samples should be isolated from the plant cell wall prior to their structural determination. Generally, analytical methods of lignin characterization are classified into two groups: destructive and nondestructive methods. The most used destructive methods include thioacidolysis (TA) [6], nitrobenzene oxidation (NBO) [7] and derivatization followed by reductive cleavage (DFRC) [8]. The nondestructive methods consist of various spectroscopic methods (e.g., UV and Fourier transform infrared spectroscopy (FT-IR spectra)) and Nuclear magnetic resonance (NMR) techniques. Details of these methods have been described in a recently published academic monograph [9]. Although significant advances were made in the area of destructive methods, the current understanding of the composition and structure of the macromolecule lignin is derived from interpretations and extrapolations of the data from degradative processes that only account for a fraction of the total polymer [10]. NMR spectroscopy has been shown to be a reliable and comprehensive method in the domain of wood chemistry. NMR spectroscopy has enormously facilitated investigations into structural aspects of complex lignin polymers. In the past, proton NMR (^1^H-NMR) was mainly used for lignin characterization; the ^1^H-NMR spectrum of acetylated lignin is used to determine the quantity of different hydroxyl groups. Lundquist and co-authors have published many works about NMR characterization of lignin, just to mention a few, which have promoted the utilization of NMR techniques in lignin characterization [11,12,13]. However, because of the polymeric nature of lignin, diversity of protons from various structures, and irregularity of linkages between building units in lignin, the ^1^H-NMR spectrum of lignin is somewhat overlapped and difficult to accurately explain. 

With the development of NMR techniques, ^13^C-NMR became popular in lignin characterization, which is a powerful way capable of revealing a large amount of lignin structural information including the presence of aryl ethers, as well as condensed and uncondensed aromatic and aliphatic carbons. Since the 1980s, many studies on ^13^C NMR spectra of lignin have been conducted [14,15,16,17]. However, the extremely low abundance of the natural ^13^C isotope makes ^13^C-NMR much less sensitive so that long acquisition times and high sample concentrations are required to enhance the sensitivity of ^13^C-NMR signals, especially for quantitative ^13^C-NMR, and thus limiting its application. Many attempts have been made to increase the sensitivity and signal-to-noise (S/N) ratios of the quantitative ^13^C-NMR spectra. Thus, quantitative ^13^C-NMR is a powerful tool in the structural analysis of lignin, especially in understanding the structural changes of lignin polymer in pulping processes and other isolation processes. 

Thanks to the rapid advances in NMR technology, it is now difficult to interpret serious structural studies on complex molecules without it. “Inverse detection” techniques can largely increase the resolution of spectra. For example, a two-dimensional heteronuclear single quantum coherence (2D-HSQC) was carried out by acquiring proton data (“Inverse detection” techniques), experiencing a 31.6-fold gain in sensitivity over the traditional carbon-detected experiment [10]. This operating method alone allows 2D ^13^C–^1^H correlation experiments to be acquired far more quickly than a 1D ^13^C NMR spectrum. Pulsed-field gradients during an NMR pulse sequence have also been accepted as normal experiments because of their capacity to refine spectra (by coherence selection) and to reduce artifacts without requiring phase cycling. Promoted by the new multidimensional quantitative NMR techniques, NMR is undoubtedly the most widely used tool for structural characterization of lignin. For example, 2D-HSQC NMR spectra attracted significant attention due to its versatility in illustrating structural features and structural transformations of isolated lignin fractions, such as milled wood lignin (MWL), cellulolytic enzyme lignin (CEL), *etc.* However, in some cases, understanding the structural changes of lignin *in situ* state (avoiding lignin separation processes) is needed. For example, a comprehensive understanding of lignin is needed when considering processes such as various pretreated techniques, including physicochemical and biological pretreatments. It should be noted that most biological processes for conversion of lignocellulosic materials to biofuels result in a vast lignin process stream, while small amounts of lignin remain in the pretreated lignocellulosic biomass, which probably affects the subsequent enzymatic hydrolysis and utilization of the biomass. Consequently, analytical techniques that permit the precise determination of the abundance and chemical attributes of the lignin (e.g., isolated form and whole cell wall form) should be developed to better understand the natural structural features of lignin and structural changes during diverse pretreatments, and further to ascertain their industrial utility. In this paper, only the advanced NMR methodologies (quantitative ^13^C-NMR and 2D-HSQC NMR techniques) are reviewed in light of their unique usefulness and popularity for characterizing lignins in an isolated and *in situ* form from lignocellulosic materials in recent years.

## 2. Solution-State NMR Methodology of the Isolated Lignin

In traditional wood chemistry, native lignin samples with less carbohydrate should be isolated prior to structural characterization of lignin in the biomass. In the past several decades, various methods have been developed to isolate native lignin from plant cell walls. The general approach to isolate native lignin involves three stages: (i) ball-milling to break up the cell wall; (ii) solvent extraction of lignin; and (iii) lignin purification. The most representative method for extracting lignin from ball milled wood was performed by aqueous dioxane (96%) treatment, named as milled wood lignin (MWL) [18], while other methods used enzymatic treatment to remove the majority of carbohydrates first, prior to solvent extraction with aqueous dioxane, resulting in cellulolytic enzyme lignin (CEL) [19]. CEL is structurally similar to MWL, but it can be obtained in a higher yield [20]. Remarkably, an improved version of the two methods has been proposed, named as EMAL [21]. The option for isolating lignin involves the use of aqueous alkaline solutions [22], especially for grass lignin. Meanwhile, to understand the delignification mechanism involved in different pretreatments, the released lignin fractions during the pretreatments were compared with the corresponding “MWL”. In addition, to understand the impact of pretreatment on the structures of lignin from pretreated substrate, the lignin from pretreated biomass was also isolated as “MWL” [23,24,25].

### 2.1. Quantitative ^13^C-NMR Techniques

#### 2.1.1. Quantitative ^13^C-NMR Spectra of non-Acetylated Lignin

**Figure 2:**
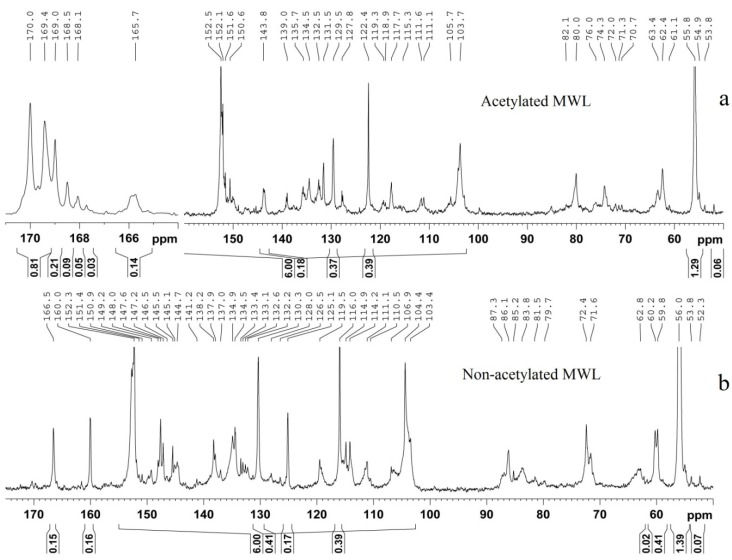
^13^C-NMR spectra of acetylated and non-acetylated bamboo lignin samples (Reprinted from [28]. Copyright 2013 De Gruyter).

Generally, both qualitative (signal assignments) and quantitative information (the relative abundance of substructures per aromatic ring) can be obtained by quantitative ^13^C NMR spectrum of non-acetylated lignin. Since the 1980s, ^13^C NMR has being used to aid in the elucidation of pulping or delignification mechanism (soda pulping, kraft pulping, and oxygen/peracid treatments), as well as pretreatments, which are discussed in detail in a recent book by Ralph and Landucci [26]. Table 1 lists an extensive compilation of structural assignments of a typical lignin (^13^C-NMR spectra of non-acetylated lignin, Figure 2b) that have been derived from previous studies [15,16,17,22,26,27]. 

**Table 1:** The chemical shift value (δ, ppm) of ^13^C-NMR spectrum of non-acetylated lignin.

ppm	assignment	ppm	assignment
166.5	C-9 in *p*-coumarate (PCE)	123.0	C-6, FE ester
160.0	C-4 in PCE	122.6	C-1 and C-6 in Ar–C(=O)C–C units
144.7	C-7 in PCE	119.4	C-6 in G units
130.3	C-2/C-6 in PCE	118.4	C-6 in G units
125.1	C-1 in PCE	115.1	C-5 in G units
116.0	C-3/C-5 in PCE	114.7	C-5 in G units
115.0	C8 in PCE	111.1	C-2 in G units
152.5	C-3/C-5, etherified S units	110.4	C-2 in G units
149.7	C-3, etherified G units	106.8	C-2/C-6, S with α-CO
148.4	C-3, G units	104.3	C-2/C-6, S units
148.0	C-3, G units	86.6	C-α in G type β-5 units
146.8	C-4, etherified G	84.6	C-β in G type β-O-4 units (*threo*)
145.8	C-4, non-etherified G	83.8	C-β in G type β-O-4 units (*erythro*)
145.0	C-4, etherified 5-5	72.4	C-γ in β-β; C-γ, β-aryl ether
143.3	C-4, non-etherified 5-5	71.8	C-α in G type β-O-4 units (*erythro*)
138.2	C-4, S etherified	71.2	C-α in G type β-O-4 units (*threo*)
134.6	C-1, S etherified C-1, G etherified	63.2	C-γ in G type β-O-4 units with α–C=O
133.4	C-1, S non-etherified; C-1, G non-etherified	62.8	C-γ in G type β-5, β-1 units
132.4	C-5, etherified 5-5	60.2	C-γ in G type β-O-4 units
131.1	C-1, non-etherified 5-5	55.6	C in Ar-OCH_3_
129.3	C-β in Ar-CH=CH–CHO	53.9	C-β in β-β units
128.0	C-α and C-β in Ar–CH=CH–CH_2_OH	53.4	C-β in β-5 units
128.1	C-2/C-6, in H units	36.8	CH_3_ group, ketones (conj) or in aliphatic
125.9	C-5/C-5' in non-etherified 5-5	29.2	CH_2_ in aliphatic side chain
122.6	C-1 and C-6 in Ar–C(=O)C–C	26.7	CH_3_ or CH_2_ group in saturated side chains
125.9	C-5, non-etherified 5-5	14.0	γ-CH_3_ in *n*-propyl side chain

Another important aspect, quantification of lignin, is very useful in lignin characterization. Prior to collection of quantitative ^13^C-NMR spectra of lignin, a number of conditions should be fulfilled. First, the lignin sample must be free of contaminants such as carbohydrates or extractives. Also, the lignin/solvent solution must be made as concentrated as possible to maximize signal-to-noise and minimize baseline and phasing distortions. Finally, the inverse-gated decoupling sequence (*i.e.*, C13IG pulse) is used which involves turning off the proton decouple during the recovery between pulses so that the Nuclear Overhauser Effect (NOE) effect is avoided.

Generally, quantitative ^13^C NMR is used only for the estimation of some specific moieties [26,29]. The most current practices in the use of quantitative ^13^C-NMR spectroscopy for the investigation of lignin are confined to using the aromatic and methoxyl signals as internal standards in expressing the various functional groups per C_9_ [26]. Such a practice is applicable to native lignin, but it is inapplicable for industrial lignin or modified lignin [29]. To overcome the above defects, Xia *et al.* suggested a novel protocol for acquiring quantitative ^13^C NMR spectra of lignins by using the internal reference compounds 1,3,5-trioxane and pentafluorobenzene [29]. The internal standards could be used for the quantification of ^13^C signals in lignins, expressed in absolute units of millimoles (mmol) per gram of sample. In addition, such quantification becomes particularly important for severely altered lignin samples. The optimum parameters for these experiments were validated for a variety of spectrometer platforms, and standard errors were determined for different spectral areas using lignin model compounds and “standard” lignins [29]. A typical quantitative ^13^C-NMR experiment is illustrated as follows: the standard program “C13IG” is selected from the program library, the inverse-gated (IG) decoupling sequence is used which involves turning off the proton decouple during the recovery between pulses so that the Nuclear Overhauser Effect (NOE) is avoided. If the concentration of lignin is more than 20% (more than 100 mg lignin in 0.5 mL DMSO-*d*_6_) then a pulse sequence: A 90° pulse width, with a 1.4 s acquisition time, and a 1.7 s relaxation delay is used. Chromium (III) acetylacetonate (0.01 M) is added to the lignin solution to provide complete relaxation of all nuclei [30]. Generally, a total of 20,000–30,000 scans are collected at 400 MHz–600 MHz NMR instruments. Subsequently, detailed approaches for the quantification of different lignin structures in milled wood lignin (MWL) have been reported by using quantitative ^13^C-NMR techniques [30,31]. Using this approach they obtained information on the lignin structure (the amount of different linkages, various phenolic/etherified noncondensed/condensed guaiacyl and syringyl moieties), which is comparable to that reported from other wet chemistry techniques, but requiring only rather short experimental times [30,31]. The results obtained for a spruce MWL were in good agreement with the vast databases for this lignin preparation and showed specific advantages of the quantitative ^13^C-NMR technique. Under the inspiration of these works, quantitative ^13^C NMR spectroscopy was used to illustrate the structural changes of lignin, isolated from various pretreated biomass, aiming to clarify the mechanism of lignin transformation during these pretreatments. For example, to investigate the effect of autohydrolysis of *Eucalyptus globulus* wood on the lignin structure, the authors selected quantitative ^13^C NMR techniques to calculate the content of β-O-4 linkages in the lignin extracted before and after autohydrolysis pretreatment [25,32,33]. The results indicated that extensive lignin degradation occurs during prehydrolysis through homolytic cleavage of the aryl-ether bonds (β-O-4 linkages). In addition, to understand the effects of acid-catalyzed ethanol organosolv pretreatment on the lignin structures of *miscanthus*, quantitative ^13^C NMR techniques were also applied [34]. The data obtained suggested that cleavage of β-O-4 linkages and of ester bonds (acetyl and coumaryl residues) were the major mechanisms of lignin breakdown during the organosolv treatment. 

#### 2.1.2. Quantitative ^13^C-NMR Spectra of Acetylated Lignin

**Figure 3:**
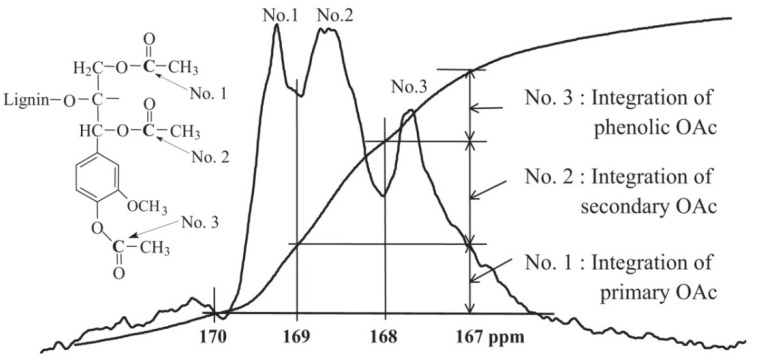
Quantitative evaluation of hydroxyl groups by ^13^C NMR spectra of acetylated lignin. (Reprinted from [36]. Copyright 2011 Elsevier).

The ^13^C-NMR spectra of acetylated lignin (Figure 2a) can be used for assigning different structures to various lignin sources. Nimz *et al.* compared structural differences between lignins of hardwoods, softwoods, and grasses by ^13^C-NMR spectra of acetylated lignin [27]. In addition, quantitative ^13^C-NMR spectra of acetylated lignin samples were also used to determine the amount of primary, secondary and phenolic hydroxyl groups [35,36]. The chemical shifts between 102 and 160 ppm were firstly set as 600, the corresponding values in the range of between δ_C_ 170–169, 169–168 and 168–167 ppm, were then obtained, which represents the amount of hydroxyl groups in the lignin (results were expressed by per/Ar) (Figure 3).

Although many works have contributed to the ^13^C-NMR spectra of lignin, there still remain some problems to be solved, such as precise signal assignments and true quantification based on ^13^C-NMR spectra of lignins, which are difficult due to signal overlap and other factors. For the signal assignments, it could be improved if advanced two-dimensional spectra were to be jointly applied. In addition, the best quantification method remains the relatively tedious inverse-gated technique, along with an internal standard substance. Fortunately, most of the semi-quantitative methods that have been described are adequate when the researcher wants to follow changes in structure during a particular treatment provided that the desired precision is maintained [26].

### 2.2. Two-Dimensional HSQC NMR Technique

With the development of modern NMR techniques, two-dimensional heteronuclear single quantum coherence (2D-HSQC) NMR experiments became popular as powerful tools for lignin characterization providing richer and less unambiguous information [37]. Assignments for lignin correlations come from the extensive database of lignin model compounds [38], along with data from a long history of NMR of both isolated and synthetic lignins [39,40,41,42,43,44,45,46,47,48,49,50,51,52,53,54]. 

#### 2.2.1. Major Structures and Aromatic Characteristics of the Isolated Lignin

**Figure 4:**
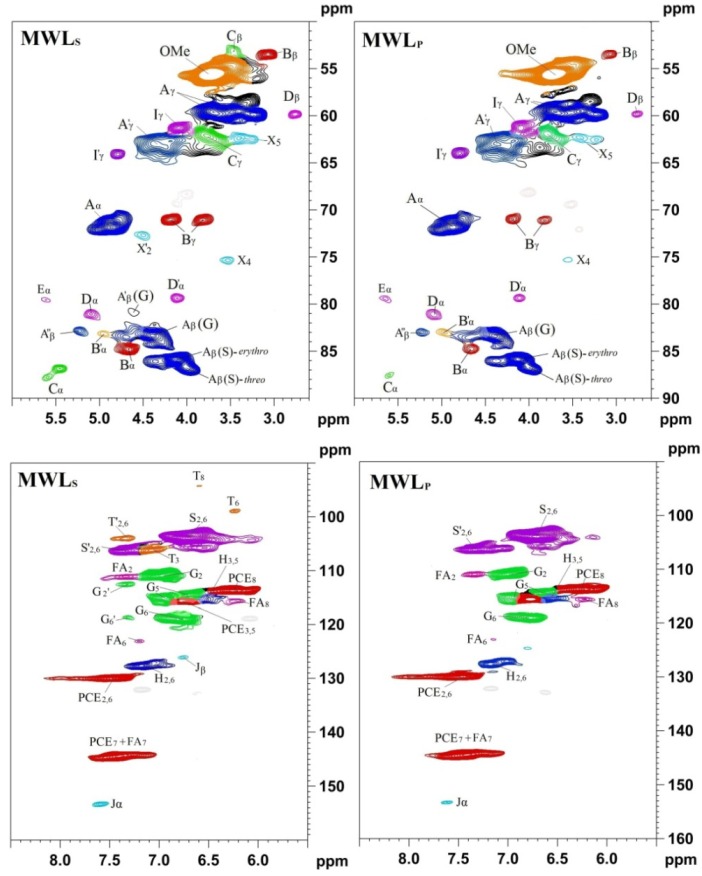
2D-HSQC NMR spectra of non-acetylated bamboo MWL specimens (MWL_S_, represents MWL extracted from bamboo stem. MWL_P_, represents MWL extracted from bamboo pith. The structural features of A, B, C, D, and E are depicted in Figure 6. (Reprinted from [28]. Copyright 2013 De Gruyter).

**Figure 5:**
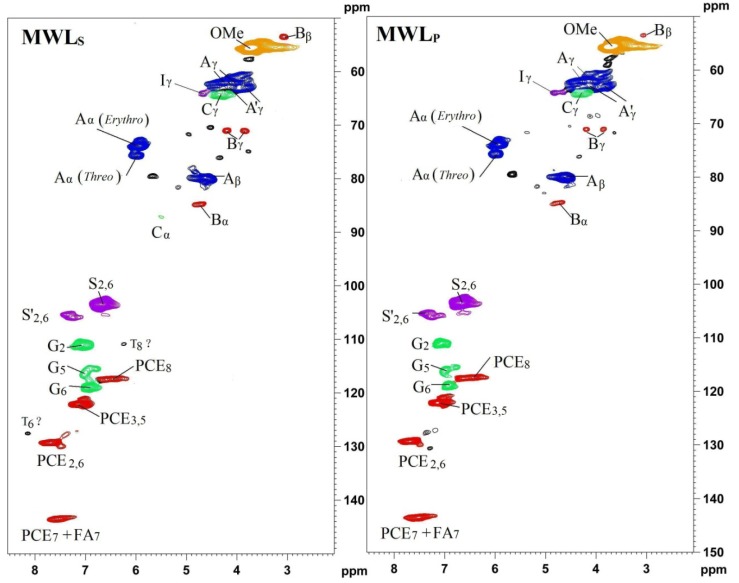
2D-HSQC-NMR spectra of acetylated bamboo MWL specimens (MWL_S_, represents MWL extracted from bamboo stem. MWL_P_, represents MWL extracted from bamboo pith. (Reprinted from [28]. Copyright 2013 De Gruyter).

**Figure 6:**
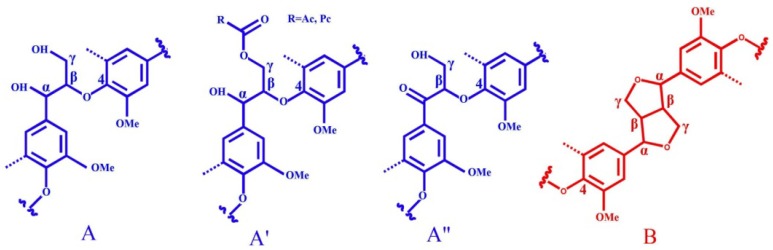

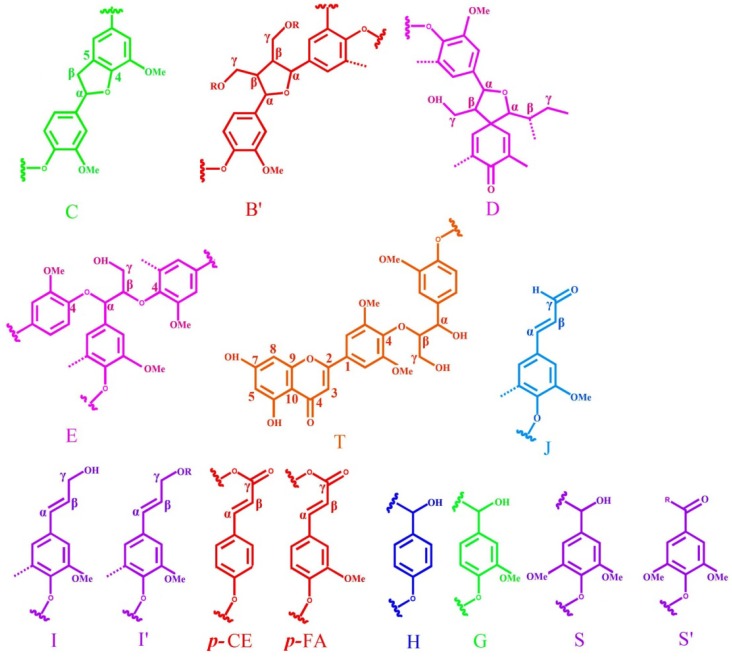
Main structures present in bamboo lignin: (**A**) β-O-4 alkyl-aryl ethers; (**A**') β-O-4 alkyl-aryl ethers with acylated γ-OH with *p*-coumaric acid; (**A**'') Cα-oxidized β-O-4' structures; (**B**) resinols; (**B**') tetrahydrofuran; (**C**) phenylcoumarans; (**D**) spirodienones; (**E**) α,β-diaryl ethers; (**T**) a likely incorporation of tricin into the lignin polymer through a G-type β-O-4 linkage; (**I**) *p*-hydroxycinnamyl alcohol end-groups; (**I**') *p*-hydroxycinnamyl alcohol end-groups acylated at the γ-OH; (**J**) cinnamyl aldehyde end-groups; (PCA) *p*-coumarates; (FA) ferulates; (**H**) *p*-hydroxyphenyl units; (**G**) guaiacyl units; (**S**) syringyl units; (**S**') oxidized syringyl units bearing a carbonyl at C_α_ (Reprinted from [28]. Copyright 2013 De Gruyter).

One advantage of 2D-HSQC NMR is that overlapping protons directly attached to carbons with different shifts are separated in the carbon dimension, whereas overlapping carbons may be separated by their direct attachment to protons in the proton dimension. Therefore, the apparent resolution of 2D spectra is much improved over that of the 1D spectrum with today’s field strengths. The 2D-HSQC experiments of non-acetylated lignin samples have been valuable in assigning major structures (β-O-4, β-β, β-5, *etc.*) in the lignin samples according to the previous studies and the above-mentioned database of lignin model compounds [37,38]. It is particularly worth mentioning that Ralph’s group at the University of Wisconsin (Madison, USA) has made great contributions in synthesizing lignin model compounds and in the identification of new structures by NMR techniques and have published many wonderful papers about lignin structures. Based on the merits of their work, the HSQC experiments have been valuable in assigning major structures of non-acetylated lignin from different origins in recent years, such as some non-woody plants [39], Jute fibers [40], bamboo [41,42,43,44,45], Triploid poplar [46,47,48], eucalyptus [49], elephant grass [50], and wheat straw [51]. The precise assignments can be found in the assigned literature references [39,40,51]. More importantly, HSQC spectra have been indispensable in identifying new and minor lignin structural units. The clear identification of dibenzodioxocins (5-5 linkages) as major new structures in lignins has been a significant finding [53,54]. Acetylated lignins (*in vitro*), are readily identified by unique and often well-resolved correlations in HSQC spectra. It is worth stressing that evidence provided by 2D NMR is far more diagnostic than 1D data purely because of the simultaneous constraints that are revealed in the data. Thus, the observation that there is a proton at 4.9 ppm directly attached to a carbon at 84.4 ppm and a proton at 4.1 ppm attached to a carbon at 82.5 ppm is more revealing than just observing two new carbons at 84.4 and 82.5 ppm in the 1D spectrum [54]. The HSQC spectra of acetylated lignins were also assigned previously [54]. For example, the detailed assignments of HSQC spectra of non-acetylated (Figure 4) and acetylated bamboo lignin samples of bamboo MWL (Figure 5) are listed in Table 2 and the major substructures are depicted in Figure 6 based on recent studies [41,45,51,52].

**Table 2:** Assignments of ^13^C–^1^H Cross signals in the quantitative heteronuclear-single-quantum-coherence spectra (HSQC) of milled wood lignin (MWL) from bamboo (Reprinted from [28]. Copyright 2013 De Gruyter).

Lable	δ_C_/δ (ppm) ^a^	δ_C_/δ (ppm) ^b^	Assignments
B′_β_	49.8/2.56	ND	C_β_–H_β_ in β-β tetrahydrofuran (B')
C_β_	53.1/3.46	ND	C_β_–H_β_ in phenylcoumaran (C)
B_β_	53.5/3.07	53.5/3.07	C_β_–H_β_ in β-β (resinol) (B)
D_β_	59.8/2.75	N.D	C_β_–H_β_ in spirodienones (D)
OCH_3_	56.4/3.70	55.6/3.76	C–H in methoxyls
A_γ_	59.9/3.35-3.80	62.0/4.08-4.27	C_γ_–H_γ_ in β–O–4 substructures (A)
A'_γ_	63.0/4.36	63.0/3.8-4.10	C_γ_–H_γ_ in γ-acylated β–O–4 (A')
C_γ_	62.2/3.76	64.3/4.33	C_γ_–H_γ_ in phenylcoumaran (C)
I_γ_	61.2/4.09	64.1/4.66	C_γ_–H_γ_ in cinnamyl alcohol end-groups (I)
I'_γ_	64.0/4.80	64.2/4.82	C_γ_–H_γ_ in cinnamyl alcohol acylated at the γ-OH (I')
B_γ_	71.2/3.82-4.18	71.7/3.84-4.20	C_γ_–H_γ_ in β-β resinol (B)
A_α_	71.8/4.86	73.8/5.93	C_α_–H_α_ in β–O–4 unit (A) (*Erythro*)
A_α_	71.8/4.86	75.6/5.96	C_α_–H_α_ in β–O–4 unit (A) (*Thero*)
A_β(G)_	83.4/4.38	76.6/5.07	C_β_–H_β_ in β–O–4 linked to G (A)
B_α_	84.8/4.66	85.6/4.70	C_α_–H_α_ in β-β resinol (B)
B'_α_	83.2/4.94	ND	C_α_–H_α_ in β-β (B', tetrahydrofuran)
A''_β_	82.8/5.23	ND	C_β_–H_β_ in β–O–4 substructures (A)
A'_β(G)_	80.8/4.62	ND	C_β_–H_β_ in β–O–4 linked to G (A)
A_β(S)_	85.8/4.12	79.8/4.63	C_β_–H_β_ in β–O–4 linked to S (A, *Erythro*)
A_β(S)_	86.7/4.00	79.8/4.63	C_β_–H_β_ in β–O–4 linked to S (A, *Thero*)
D_α_	81.0/5.10	ND	C_α_–H_α_ in spirodienones (D)
D'_α_	79.4/4.10	ND	C'_α_–H'_α_ in spirodienones (D)
E_α_	79.6/5.60	ND	C_α_–H_α_ in α,β-diaryl ethers (E)
C_α_	86.8/5.45	87.1/5.49	C_α_–H_α_ in phenylcoumaran (C)
T'_2,6_	103.9/7.34	ND	C'_2,6_–H'_2,6_ in tricin (T)
T_6_	98.9/6.23	ND	C_2,6_–H_2,6_ in tricin (T)
T_8_	94.2/6.60	ND	C_8_–H_8_ in tricin (T)
T_3_	106.2/7.07	ND	C_3_–H_3_ in tricin (T)
S_2,6_	103.9/6.70	103.5/6.66	C_2,6_–H_2,6_ in syringyl units (S)
S'_2,6_	106.3/7.32	105.4/7.37	C_2,6_–H_2,6_ in oxidized S units (S')
G_2_	110.8/6.97	111.0/7.07	C_2_–H_2_ in guaiacyl units (G)
G_5_	114.5/6.70	116.5/7.00	C_5_–H_5_ in guaiacyl units (G)
G_5e_	115.1/6.95	ND	C_5_–H_5_ in etherified guaiacyl units (G)
G_6_	119.0/6.78	118.9/6.90	C_6_–H_6_ in guaiacyl units (G)
J_β_	126.1/6.76	ND	C_β_–H_β_ in cinnamyl aldehyde end-groups (J)
H_2,6_	127.7/7.17	127.8/7.34	C_2,6_–H_2,6_ in H units (H)
PCE_3,5_	115.6/6.77	122.1/7.14	C_3,5_–H_3,5_ in *p*-coumarate (PCE)
PCE_2,6_	130.2/7.48	129.3/7.68	C_2,6_–H_2,6_ in *p*-coumarate (PCE)
PCE_7_	144.8/7.51	143.5/7.52	C_7_–H_7_ in *p*-coumarate (PCE)
PCE_8_	113.7/6.24	117.4/6.45	C_8_–H_8_ in *p*-coumarate (PCE)
FA_2_	110.7/7.35	ND	C_2_–H_2_ in ferulate (FA)
FA_6_	123.1/7.20	ND	C_6_–H_6_ in ferulate (FA)
FA_7_	144.8/7.51	ND	C_7_–H_7_ in ferulate (FA)
J_α_	153.4/7.59	ND	C_α_–H_α_ in cinnamyl aldehyde end-groups (J)

^a^ δ_C_/δ_H_ (ppm), the chemical shift (ppm) of non-acetylated sample; ^b^ δ_C_/δ_H_ (ppm), the chemical shift (ppm) of acetylated sample; ND: Not detected; Signals were assigned by comparison with the literatures [39,40,41,51,52,54].

#### 2.2.2. Lignin-Carbohydrate Complex (LCC) Linkages

The major inter-unit linkages within the lignin monomer (G, S, and H) are β-O-4, β-β, β-5, β-1 substructures. Besides the inter-unit linkages, lignin also associates with carbohydrates via various chemical linkages, which restricts the efficient separation of lignin from plant cell wall. Thus, it is important to understand the LCC linkages of lignin samples. The main types of lignin-carbohydrate complexs (LCC) in wood are believed to be phenyl glycoside bonds, esters, and benzyl ethers [55]. Most of the information on lignin and LCC structures was obtained previously from different wet chemistry techniques and model compound experiments [55]. Although the information obtained from these methods is very valuable, each method gives limited information and is not able to provide a general picture of the entire lignin and LCC structures [48]. 

##### ***Benzyl*** ***ether LCC***

Benzyl ether LCC structures can be subdivided as follows: (a) C1-linkages between the α-position of lignin and primary OH groups of carbohydrates (at C-6 of Glc, Gal and Man and C-5 of Ara) giving a cross-peak at 80–81/4.5–4.7 ppm and (b) C2-linkages between the α-position of lignin and secondary OH groups of carbohydrates, mainly of lignin-xylan type, giving a cross-peak at 80–81/5.1–4.9 ppm [55]. The signal of lignin–xylan benzyl ether linkages (C2) is overlapped with the signal of spirodienone lignin moieties (D, shown in Figure 4) at 81.2/5.10 ppm.

##### ***Esters*** ***LCC Linkages***

Generally, benzyl ester (α-ester) structures were detected at δ_C_/δ_H_ 75/6.1 ppm in the HSQC spectra [44] and the signals of CH_2_-γ in γ-esters were observed in the area of δ_C_/δ_H_ 65–62/4.0–4.5 ppm. However, a possibility of overlapping LCC γ-esters signals with those of various types of lignin γ-esters, such as benzoate, coumarate, and acetate lignin moieties, should be considered [56,57]. For example, *Populus* species contain *p*-benzoates structures [5], non-wood lignins contain ferulate and coumarate derivatives [5], grass lignin (maize lignin) contain *p*-coumarate structures [58], bamboo lignin contains amounts of *p*-coumarate structures at γ-position of the lignin side-chain [45]. The investigated pine and birch preparations do not contain these types of ester moieties such as *p*-benzoates structures and *p*-coumarate structures and therefore could be used for investigating the ester LCC Linkages, such as γ-ester [57]. 

##### ***Phenyl glycoside*** ***(PhGly) LCC Linkages***

**Figure 7:**
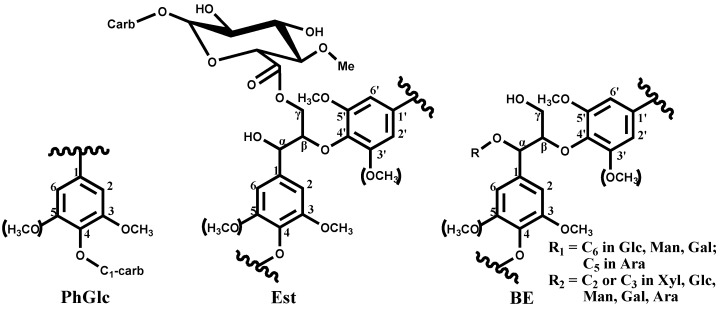
Main lignin-carbohydrate linkages: PhGlc, phenyl glycoside; Est, γ-ester; and BE, benzyl ether (Reprinted from [48]. Copyright 2012 American Chemical Society).

Phenyl glycoside linkages give a group of signals of carbohydrates C-1 at 104–99/4.8–5.2 ppm according to model compound data [55]. A variety of signals indicate the involvement of different types of carbohydrates (different sugars and different acetylation mode, especially for xylan) in phenyl glycoside linkages. Hitherto, only rare literature has focused on the quantitative information of these LCC linkages [57]. Recently, Yuan *et al.* isolated some lignin fractions with a high content of associated carbohydrates and this provides adequate and quantitative information for the entire lignin structure as well as various LCC linkages at the same time using a newly developed quantitative NMR method by Zhang *et al.* [48,59]. The various LCC linkages were found in poplar wood and the structures are shown in Figure 7 [48]. 

### 2.3. Quantification of Lignin Structures by NMR Techniques

Besides the qualitative assignments of the lignin structures, quantitative measurement of the lignin structures is another important aspect for investigating lignin. Generally, quantitative measurement of various structures of lignins is possible when appropriate standards or pulse sequences are applied [39,59,60,61,62,63,64]. It has been suggested that a Q-HSQC NMR pulse sequence could be used to suppress the ^1^J_C–H_ dependence of the HSQC NMR signals [59]. Nevertheless the Q-HSQC sequence still fails to quantify structures in polymeric samples, since it does not solve the errors caused by T2 relaxations and resonance offsets [60]. Zhang and Gellerstedt [60] presented a new analytical method based on the 2D-HSQC NMR sequence and quantitative ^13^C-NMR, which can be applied for quantitative structural determination of complicated polymers, such as lignin and cellulose derivatives. The key to this method is selecting the proper internal standard reference signal (s) to eliminate the major errors caused by T2 relaxations, resonance offsets, coupling constant deviations and homonuclear couplings. The suitable internal standard reference signal definitely originated from lignin with similar structural features. The selected internal standard references can convert relative integration values obtained from the corresponding 2D spectrum to absolute values coupled with the quantitative ^13^C NMR spectrum. Recently, a very popular pulse program used in 2D-HSQC quantification is the adiabatic pulse sequence, named as hsqcetgpsisp.2, which means phase-sensitive 2D-HSQC using echo-antiecho and adiabatic pulses for inversion and refocusing, can be selected from Bruker pulse program library [62,63,64]. This has the advantage of J-independence and offset insensitivity over an essentially unlimited active bandwidth. The program is less sensitive to differences in one-bond ^13^C–^1^H coupling constants and the response over the entire spectral range is more uniform, suggesting that improved quantification should result [62]. Besides, other pulse programs have also been described in the classic literature, which focus on solution state NMR in lignin [37]. 

Quantitative strategy is another important aspect in lignin quantification. According to the literature, “semi-quantitative and quantitative” strategies were adopted in NMR characterization of lignin, which depends on the internal standard (IS) selected, such as the aromatic units and methoxy groups. 

#### 2.3.1. Relative Quantitative Method Based on 2D-HSQC Spectra (without IS)

The most used strategy is a semi-quantitative method based on 2D-HSQC spectra without an internal standard [39,41], which as the name implies, is a relative quantitative method. In other words, the results obtained cannot be used to compare the absolute differences between the diverse lignin fractions since “normalization” was used in this method. The typical method to calculate the relative abundance of diagnostic structures is described as follows, relative percentages of A–E units (Figure 4) were estimated via a semi-quantitative analysis of the volume integral of the HSQC cross-signal by the following formula:

I*_X_*% = I*_X_*/(I_A_ + I_B_ + I_C_ + I_D_) × 100%

where I_A_, I_B_, I_C_, and I_D_ are the integral values of α-position of β-O-4 (A), β-β (B), β-5(C), and β-1(D), respectively. (All of the integration should be conducted at the same contour level.) 

#### 2.3.2. Quantitative Method Based on 2D-HSQC Spectra (IS: Aromatic Units) 

Besides the semi-quantitative strategy, another quantitative strategy is based on HSQC spectra, which selected “aromatic units” as IS. Particularly, the method uses a cluster of signals that are representative of all C9 units, *i.e.*, IS. The choice of the G_2_, S_2,6_/2 + G_2_, and 0.5IS_2,6_ + IG_2_ + 0.5IH_2,6_ signals as IS are for softwood [61], hardwood lignin [61], and grass lignin [65], respectively. The results were expressed as how much linkage (<1.0) per aromatic ring.

IC9 units = 0.5IG_2_ (softwood lignin) IC9 units = 0.5IS_2,6_ + IG_2_ (hardwood lignin) IC9 units = 0.5IS_2,6_ + IG_2_ + 0.5IH_2,6_ (grass lignin)

Where IS_2,6_ is the integration of S_2,6_, including S and S', IG_2_ is the integral value of G_2_. IH_2_ is the integral value of H_2,6_. IC9 represents the integral value of the aromatic ring. According to the internal standard (IC9), the amount of I_X_% could be obtained by the following formula,

I*_X_*% = I*_X_*/I_C9_ × 100%

Where I*_X_* is the integral value of the α-position of A (β-O-4), B (β-β), C (β-5), and D (β-1), the integration should be in the same contour level.

#### 2.3.3. Quantitative Method Based on the Combination of ^13^C-NMR and 2D-HSQC Spectra

The method, proposed by Zhang and Gellerstedt [60], is a combination of HSQC and quantitative ^13^C NMR techniques, which gives more reliable data about the lignin linkages in the lignin samples:

I*_X_*% =2I*_X_*/2I (90−78/6.0−2.5) × ^13^C (90.0−78.0)/^13^C (163.0 − 103.6) × 600

Where I*_X_* is the integration of A_β_ (both A_β_ (G/H) and A_β_(s)), B_α_, C_α_, D_α_ in the region of 90−78/6.0−2.5, I (90−78/6.0−2.5) is the total integration of this region (should have the same contour level as A, B, C, and D units). The key principle in the quantification method is based on “absolute quantification by ^13^C-NMR spectra, relative quantification by 2D-HSQC spectra (distinguished lignin units from overlapped signals with carbohydrates)”. Generally, the integral values for the total aromatic carbons (163.0–103.6 ppm) were set as 600.0. The integral values for the selected IS references and other structural moieties are expressed per 100 Ar. Based on the quantitative method (Section 2.3.3), quantitative values for different structures were obtained [45,48,60]. 

#### 2.3.4. Quantitative Method Based on the Combination of NMR Spectra and the Methoxy Integration Method

The quantification strategy is based on the lignin methoxyl (OMe) integration method. Generally, the determination of the lignin methoxyl content is based on an iodometric method, which can determine the molar quantity of OMe per gram of original wood [66]. In addition, the detailed procedure of the quantitative method was carried out as follows: 1). The absolute content of β-O-4, β-β, and β-5 (per aromatic ring) was first quantified by ^13^C-NMR spectrum: 2). The integral for C_α_–H_α_ correlation of each structure was divided by the OMe integral, and the resulting ratio was multiplied by the iodometrically determined molar quantity of OMe per gram of original wood [67,68]. If some substructures could not be accurately integrated from ^13^C-NMR spectrum of lignin, the 2D-HSQC spectrum of lignin was needed under the circumstances. For example, the integral for the C_α_–H_α_ of the phenylglycerol was divided by the integral for the C_α_–H_α_ cross-peak of the β-O-4 units, and the resulting ratio was multiplied by the previously calculated molar quantity of β-O-4 units per gram of original wood (*i.e.*, per 1.0 g for control wood and per 0.303 g for decayed wood) [68]. Therefore, the absolute content of phenylglycerol was obtained. 

Another method based on this strategy, developed by Yelle *et al.* [69], used quantification of the lignin substructures identified by *in situ* HSQC technique. At first, the methoxyl content (millimoles of lignin methoxyls (OMe) per gram of original wheat straw) was calculated based on the chemical composition analysis. Methoxyl content was assumed constant throughout the hydrothermal and enzymatic hydrolysis treatments. Next, the HSQC NMR spectra of different wheat straw-treated samples were used to determine the β-aryl ether, *O*-acetyl, and uronic acid content. More specifically, the integral for the ^13^C–^1^H correlation for the β-aryl ether α-C/H (A_α_), the integral for the ^13^C–^1^H correlation for the acetate methyl (–CH_3_), the two integrals for the ^13^C–^1^H correlations for the acetylated xylan structures (2-*O*-Ac-Xylp and 3-*O*-Ac-Xylp), and the integral for the ^13^C–^1^H correlation for the anomeric position of 4-*O*-MeGlcA was divided by the integral for the ^13^C–^1^H correlation for the OMe, and the resulting ratio was multiplied by the determined molar quantity of OMe per gram of original wheat straw to obtain the quantitative results of different lignin samples. 

## 3. Characterization of Lignin via *in-situ* 2D-HSQC NMR Methodology

Qualitative and quantitative characterization is important for understanding the detailed structural features of isolated lignin, which will eventually contribute to the goal of developing lignin-based value-added products in the future. However, in some cases, the structural changes of lignin should be investigated by an *in* *situ* 2D-HSQC NMR approach (without lignin isolation). For example, we need to *in situ* monitor structural changes of components (especially for lignin) in lignocellulosic biomass during various chemical or biological processes in the biofuel and chemical production. However, heterogeneous complexity and low solubility are two of the most challenging biomass characteristics that hinder rapid NMR characterization of lignin via *in situ* state. Generally, prior to *in* *situ* 2D-HSQC NMR characterization, ball-milling, dissolution of plant cell wall materials (e.g., chemical or biological treated cell wall) was needed. Up to now, there have been two available approaches to achieve the goal of direct NMR characterization of biomass. (1) Whole cell wall dissolved system [dimethylsulfoxide/*N*-methylimidazole (DMSO/NMI, 2:1)] was used to dissolve the cell wall or treated cell wall followed by acetylation, precipitation into water, freeze-drying, and then dissolution in CDCl_3_ or DMSO-*d*_6_; (2) The swelling of the ball-milled cell wall material (*i.e.*, without acetylation) carried out directly in the DMSO-*d*_6_, DMSO-*d*_6_/pyridine-*d*_5_ and perdeuterated pyridinium chloride-DMSO system. 

### 3.1. Solution-State 2D-HSQC NMR of Acetylated Plant Cell Walls in a Whole Cell Wall Dissolving System (DMSO/NMI and Ionic Liquid)

For a long time, researchers have been searching for methods that can analyze the lignin *in situ* state since traditional isolation and purification methods generally alter the lignin structure to some extent. Fortunately, solution-state NMR techniques coupled with appropriate dissolution or gelling solvents, can achieve the *in-situ* characterization of lignin. The concept of whole plant cell wall characterization by solution-state 2D-HSQC NMR was firstly proposed by Lu and Ralph, who developed a bisolvent system of *N*-methylimidazole (NMI) and dimethyl sulfoxide (DMSO) to dissolve and acetylate finely ball-milled plant cell walls and performed detailed structural studies on the acetylated plant cell-wall solutions using high-resolution NMR spectroscopy [70]. Although the correlations in the spectra were expectedly dominated by polysaccharides, the well-dispersed signals allowed substantive assignments to various structures of cellulose, hemicelluloses and lignin. For instance, although lignin present in whole cell walls is of relatively low content, most of its structural features are evident and correlations from the aromatic region, provides very useful information about the syringyl/guaiacyl composition of entire lignin, which cannot be obtained by any other degradative methods [70]. Although the initial results cannot meet the elaborate requirements of characterizing the lignin polymer in the cell wall, the idea stimulated more researchers to do more detailed work to realize the *in-situ* status lignin characterization by solution-state 2D NMR. For instance, under the enlightenment of the bisolvent system, Holtman *et al.* applied the DMSO-NMI solvent system to prepare several acetylated lignin samples, ball-milled wood, milled wood lignin (MWL), and residual lignin (RL), from the sapwood of Loblolly pine (*Pinus taeda*) [71]. All samples were treated with industrial cellulase to remove the carbohydrates before dissolution in the DMSO/NMI system and then acetylation. Results from this work suggested that the residual enzymatic lignin (REL) is closer in structure to the original lignin than the MWL and the residues after cellulase treatment are readily acetylated in the DMSO/NMI solvent system, which may be the most suitable preparation for lignin characterization by NMR techniques [71].

**Figure 8:**
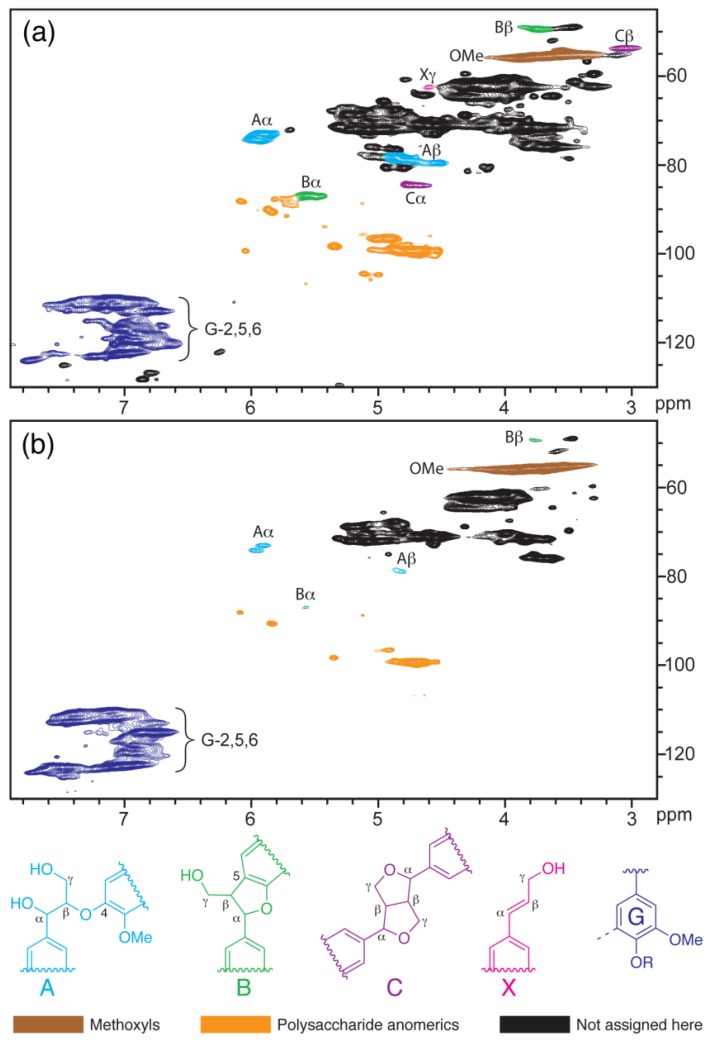
HSQC NMR spectra of acetylated sound spruce wood (**A**) and acetylated, brown-rotted spruce wood (**B**) in DMSO-*d*_6_. Most of the unassigned structures are attributable to non-anomeric carbon–hydrogen bonds in polysaccharides. OMe: methoxyl. R = lignin polymer or H (Reprinted from [67]. Copyright 2008 Wiley).

Another successful example is monitoring the lignin changes during the biodegradation process via *in situ* HSQC NMR by using this solvent system (DMSO/NMI). It is generally thought that brown-rot basidiomycetes do not degrade lignin significantly because the “lignins” left in decayed wood remain [67]. The DMSO/NMI solvent system was applied to dissolve and acetylate brown-rotted wood to allow examination of the structural changes of lignin in the wood by Yelle *et al.* [67] (Figure 8). Compared to the fresh wood, *in situ* 2D-HSQC solution-state NMR spectra showed that brown rot decay by *Gloeophyllun trabeum* in spruce wood led to remarkable non-selective degradation (cleavage) of linkages between structural units in lignin, although most of its aromatic residues were retained [67]. Similarly, aspen wood degraded by *Postia placenta* was also investigated by *in* *situ* HSQC NMR methods. The results showed that decay decreased the content of β-O-4 linkages in the lignin by more than half, while greatly increasing the relative content of several truncated lignin structures in decayed wood as compared to sound wood [68].

**Figure 9:**
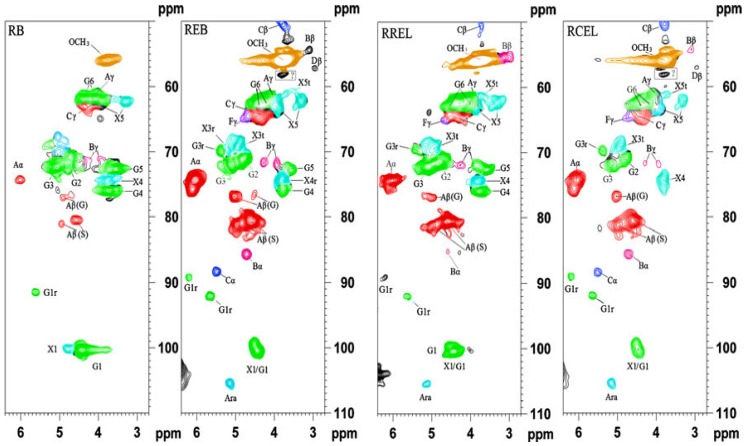
The side chain region of HSQC spectra of the acetylated bamboo samples (RB, regenerated bamboo from DMSO/NMI solvent system; REB, residue after enzymatic hydrolysis of RB; RCEL, dioxane lignin extracted from REB; RREL, residue after RCEL extraction from REB. The lignin structures identified are also shown: (**A**) β-O-4' substructure; (**B**) resinol substructure; (**C**) phenylcoumaran substructure; (**F**) *p*-hydroxycinnamyl alcohol end groups; (**G**) β-glucan; (**X**) xylan (**A**) arabinose (Reprinted from [65]. Copyright 2012 SpringerLink).

Logical application of the DMSO/NMI solvent system includes predicting biomass processing efficiency as well as for optimizing pretreatment methods in various biorefinery processes. For example, *in situ* 2D-HSQC NMR characterization of the structural heterogeneity of lignin polymers during successive treatment of bamboo was emphatically performed in a recent study [65]. The heterogeneous lignin polymers during the pretreatment in DMSO/NMI and enzymatic hydrolysis were *in-situ* tracked by the HSQC NMR technique. The study showed that all the lignin polymers in the differently treated bamboo were demonstrated to be HGS-type and partially acylated at the γ-carbon of the side chain by *p*-coumarate and acetate groups. In addition, the major lignin linkages (β-O-4, β-β, and β-5, *etc.*) and various lignin-carbohydrate complex linkages (benzyl ether and phenyl glycoside linkages) can be assigned (Figure 9), and the frequencies of the major lignin linkages were quantitatively obtained by the method aforementioned. In particular, the residual enzyme lignin (REL) contained a higher amount of syringyl units and less condensed units as compared to other samples. The method gives us a vision to track the structural changes of plant cell walls (e.g., lignin polymers) during the different pretreatments [65].

In addition to the DMSO/NMI solvent system, ionic liquid ([Bmim]Cl) was also used as reaction medium to acetylate cell walls for *in* *situ* 2D-HSQC NMR characterization. The *in* *situ* HSQC NMR technique of acetylated cell wall was also applied to detect the lignin changes between archaeological wood and fresh wood [72,73]. Afterwards, Qu *et al.* identified the whole cell-wall components (including lignin, cellulose, and hemicelluloses) by the 2D-HSQC NMR technique, with the aid of isolated lignin and commercial cellulose and hemicelluloses (arabinoxylan, galactomannan, and glucomannan) [74]. However, the above-mentioned chemical modification of the plant cell wall, with even simple derivatization, led to the loss of some structural information. For example, natural acetylation in the plant cell wall was masked when the sample was per-acetylated.

### 3.2. Solution-State 2D-HSQC NMR of non-Acetylated Plant Cell Walls in Deuterated Solvent

**Figure 10:**
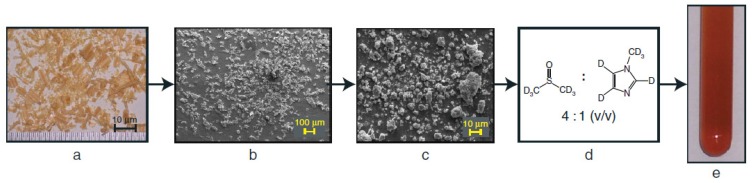
A diagram illustrating the nonderivatized dissolution of plant cell walls. *Pinus taeda* is shown here as an example: (**a**) shavings produced via a conventional planer; (**b**) cryogenically mixer-milled; (**c**) planetary ball-milled; (**d**) dissolution method; (**e**) NMR tube of cell walls in solution (Reprinted from [75]. Copyright 2008 Wiley).

Ideally, “native” whole plant cell walls could be directly characterized by solution-state 2D-HSQC NMR (*in* *situ* state) if the whole cell wall can be dissolved in deuterated or mixed solvents (Figure 10) [75]. It was noted that Yelle *et al.* were the first to introduce a mixture of deuterated solvents (DMSO-d_6_/NMI-d_6_) to investigate the entire wood cell wall without acetylation. Fortunately, through this approach native acylations were revealed [75]. 

Thanks to the different whole cell wall dissolved systems found by researchers, the *in* *situ* 2D-HSQC NMR characterization of the plant cell wall could be realized with satisfactory results via advanced NMR equipment (400 MHz or higher). Structural characterization of lignin could be directly investigated by NMR investigations via non-derivatization solvent systems, such as DMSO-*d*_6_/NMI-*d*_6_ [75], DMSO-*d*_6_ [76] and DMSO-*d*_6_/pyridine-*d*_5_ [77]. The development of DMSO-*d*_6_/NMI-*d*_6_ was to enhance the peak dispersion of the polysaccharides and to enable visualization of natural acylations in woody plants since NMI facilitates dissolution of cellulose and xylan/mannan [75]. However, some overlapping solvent signals still mask portions of the lignin aromatic region. Because of this, the DMSO-*d*_6_ method has been developed subsequent to the DMSO-*d*_6_/NMI-*d*_6_ work to use NMR as a rapid screening process for lignin chemistry. 

**Figure 11:**
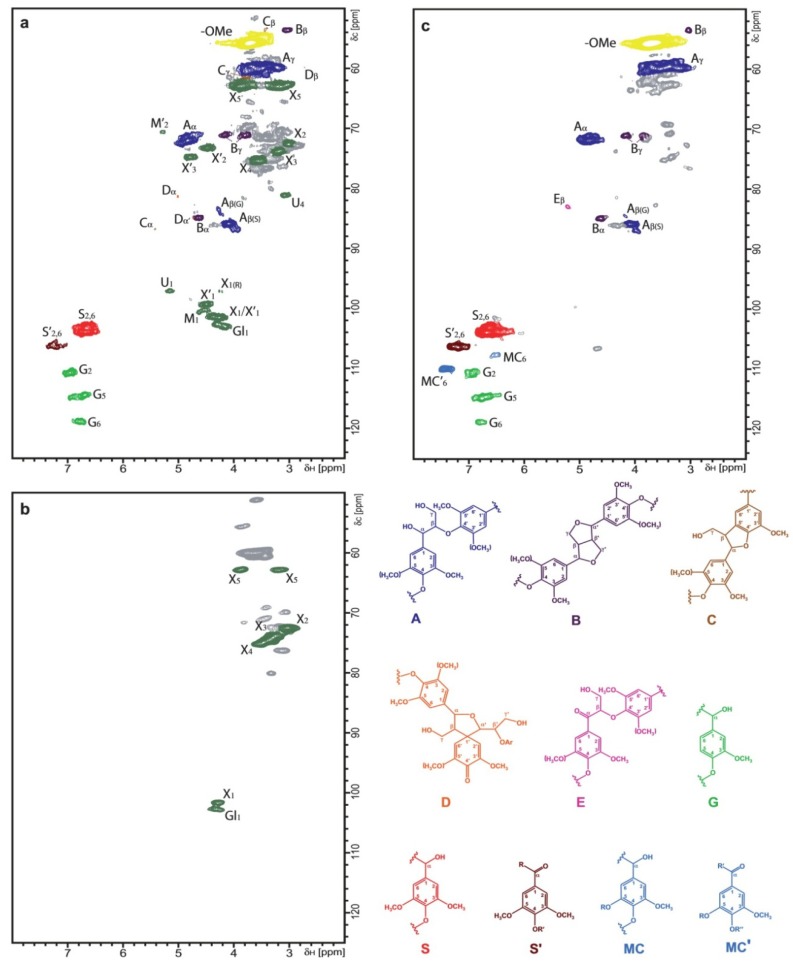
Two-dimensional (2D) NMR spectra (HSQC experiments) at the gel state of: (**a**) sound *E. cordifolia* wood; (**b**) *E. cordifolia* wood after selective white-rot fungal decay under environmental conditions (by G. australe); and (**c**) *E. cordifolia* wood after selective brown-rot fungal decay under environmental conditions. The lignin structures identified are also shown: (**A**) β-O-4' substructure; (**B**) resinol substructure; (**C**) phenylcoumaran substructure; (**D**) spirodienone substructure; (**E**) C_α_-oxidized substructure A; (**G**) guaiacyl unit; (**S**) syringyl unit; (**S**') C_α_-oxidized S unit (R, lignin or OH; R′, H or lignin); (**MC**) 5-hydroxyguaiacyl (R, H) or 5-aryl-ether-guaiacyl unit (R, lignin aromatic ring); (**MC**') C_α_-oxidized MC unit (R', lignin or OH; R'', H or lignin); The carbohydrates identified are also shown (X) β-xylan units; (**U**) α-glucuronic acid; (**G1**) β-glucan (Reprinted from [81]. Copyright 2011 Wiley).

The rapid NMR characterization method provides what appears to be the best tool for the detailed structural study of the complex cell wall polymers. At the same time, the above-mentioned solvent systems were also used to detect the lignin polymers from various origins including artificially lignified cell walls [78], hardwood (*Eucalyptus globulus*) [79], softwood (*Picea abies*), and non-woody plants (*Agave sisalana*) [80], as well as *E. cordifolia* wood after selective white-rot fungal decay and brown-rot fungal decay [81]. A typical procedure for *in-situ* characterizing the lignin polymer in treated wood is as follows: 100 mg of finely ball-milled wood (fresh or treated) was suspended in 0.75 mL of DMSO-*d*_6_ in the NMR tube and sonicated for 10 min in an ultrasonic bath, after spectra collection, 2D-HSQC NMR spectra (Figure 11) of the white-rotted wood showed only cellulose and (deacetylated) hemicelluloses, and the complete removal of lignin. On the other hand, the brown-rotted wood showed the nearly complete absence of polysaccharides, while the main features of lignin structure, as revealed by 2D-HSQC NMR spectra, could be distinctly observed. These included well-resolved aromatic and side-chain cross-signals, although the intensity of the latter signals based on aromatic unit basis (obtained by method listed in Section 2.3.2) was lowered indicating a reduction in the number of side-chain linkages (β-O-4 and β-β) per aromatic unit. Although the relative abundances (obtained by the method listed in Section 2.3.1) remained unchanged. However, a comparison of the lignin side-chain (involved in β-O-4', β-β' and other linkages) and methoxyl cross-signals on an aromatic unit basis indicates around 45% depletion of the above linkages and near 20% depletion of methoxyls. 

**Figure 12:**
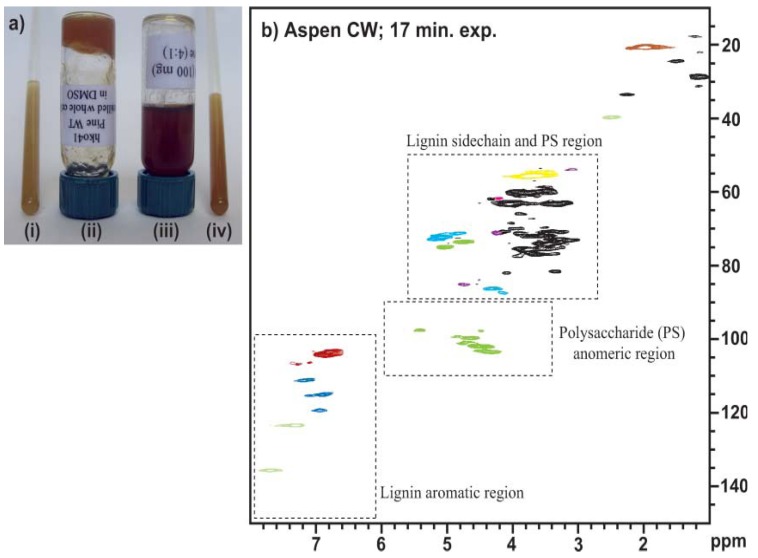
(**a**) Gel-samples of ball-milled cell walls; (i) gel-sample (~70 mg) in a 5 mm NMR tube in 1 mL of DMSO-*d*_6_; (ii) a gel-sample in DMSO in a vial (upside down to demonstrate its viscosity); (iii) a DMSO/pyridine (4:1) gel-sample in a vial demonstrating its improved mobility; (iv) gel-sample (~70 mg) in a 5 mm NMR tube in 1 mL of DMSO-*d*_6_/pyridine-*d*_5_ (4:1); (**b**) Short acquisition time (17 min) experiment on aspen cell walls in DMSO-*d*_6_/pyridine-*d*_5_ (4:1) using a 750 MHz cryoprobe-equipped NMR. The spectrum is adequate for many purpose-chemometrics, and S/G ratio estimation, for example (Reprinted from [77]. Copyright 2008 Wiley).

Among the above-mentioned deuterated solvents system, DMSO-*d*_6_/pyridine-*d*_5_ was deemed as the most promising solvent. For example, with DMSO-*d*_6_/pyridine-*d*_5_, a 17 min experiment (Figure 12) using a 750 MHz cryoprobe-equipped NMR instrument provided a spectrum which is adequate for most purposes, such as chemometrics and S/G ratio estimation. This result clearly shows that the instrument can acquire a satisfactory 2D-HSQC NMR spectrum of the whole cell wall in 1 h, implying that acquiring spectra is not the barrier to obtaining data for over 20 samples per day [77]. With the new potential for chemometric analysis using the 2D-HSQC NMR fingerprint, this gel-state method may provide the basis for an attractive approach to providing a secondary screen for selecting biomass lines and for optimizing biomass processing and conversion efficiencies [77]. However, the cellulose contours are underrepresented in *in* *situ* 2D-HSQC NMR of non-acetylated cell wall since the crystalline cellulose is not fully swollen, despite the rather clear appearance of the solution. The dissolved cell wall in the NMR tube provides a gel that permits spectra with reasonable dispersion and resolution to be acquired. At the same time, as the samples are not acetylated, native cell wall acetylation is easily detected, and the polysaccharide anomeric signals are often more dispersed. In addition, the absence of cellulose signals also facilitates the identification of lignin and hemicelluloses. With this improved solvent, several plant cell wall samples were examined by 2D-HSQC NMR spectroscopy to demonstrate its better features compared to the original method using DMSO-*d*_6_ alone as gelling solvent [77]. It should be noted that the cellulose signals are not the only signals suppressed with the DMSO-*d*_6_ (gel-state) method, xylan and mannan are both highly polymeric and also undoubtedly suppressed as well. Therefore, the DMSO-*d*_6_ (gel-state) method was streamlined for lignin analysis, not polysaccharides analysis. However, based on these non-acetylated solvent systems, lignin composition (notably, the syringyl:guaiacyl: *p*-hydroxyphenyl ratio) could be quantified without the need for lignin isolation. Correlations for *p*-coumarate units and simple ferulates in the corn sample are readily seen and well resolved. 

**Figure 13:**
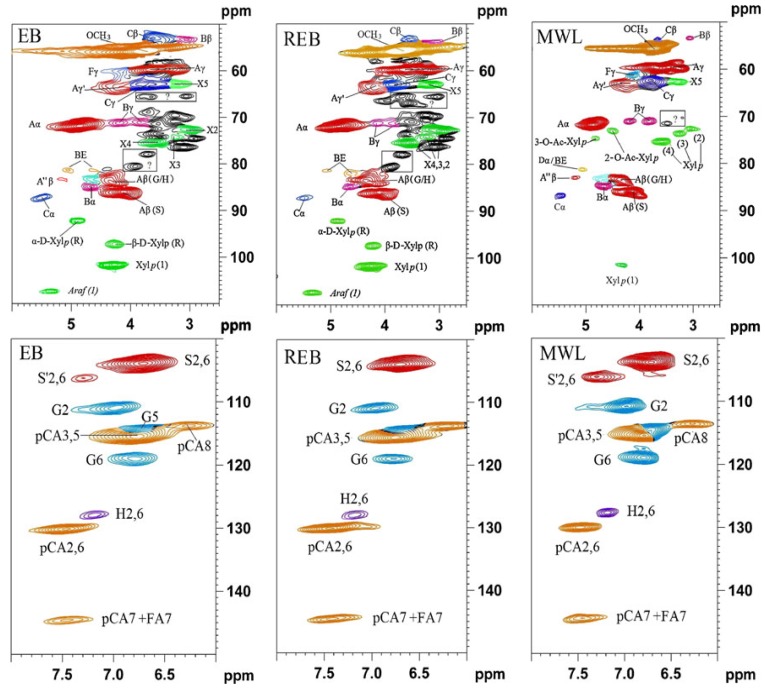
HSQC spectra of non-acetylated EB, REB, and MWL (directly dissolved in DMSO-*d*_6_, EB, bamboo having undergone enzymatic hydrolysis; REB, regenerated bamboo having undergone enzymatic hydrolysis; MWL, milled wood lignin from bamboo) (Reprinted from [65]. Copyright 2012 SpringerLink).

A recent study showed that 2D-HSQC NMR spectra of enzymatic hydrolysis residue (EHR) provided remarkably well resolved spectra that can be compared to that of MWL [65]. The structural features and distribution of inter-unit linkages of the EHR could be assigned and quantified (Figure 13). The major substructures, such as β-ether (β-O-4) A, resinol (β-β) B, and phenylcoumaran (β-5) C can be readily assigned in non-acetylated EHR samples. Besides, these substructures were quantified based on the method mentioned in Section 2.3.2. Based on the results obtained, it was found that *in situ* characterization of pretreated biomass by HSQC NMR analysis is a beneficial structural analysis methodology in the emerging biomass research field for the characterization of enzymatic hydrolysis residues (EHR).

**Figure 14:**
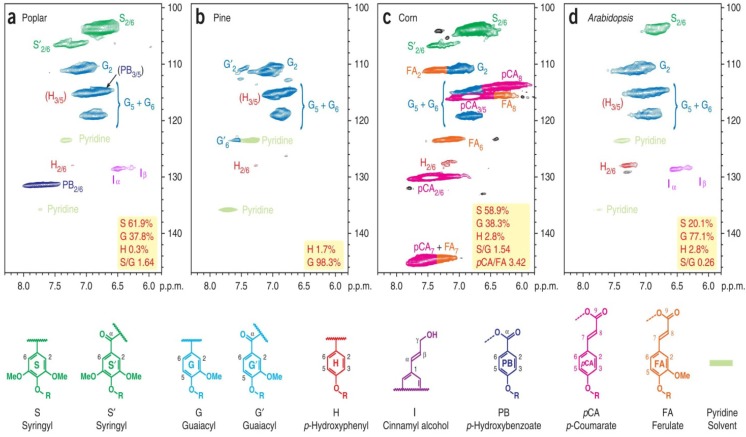
2D-HSQC NMR spectra revealing lignin unit compositions. 2D-HSQC spectra (aromatic regions only) of cell wall gels in 4:1 (vol/vol) DMSO-*d*_6_/pyridine-*d*_5_ from (**a**) 2-year-old greenhouse-grown poplar wood; (**b**) mature pine wood; (**c**) senesced corn stalks and (**d**) senesced Arabidopsis inflorescence stems. Contours in this region are used to measure S/G/H ratios, as well as relative *p*-hydroxybenzoate (PB, in poplar), *p*-coumarate (PCA in corn) and ferulate (FA in corn) levels (Reprinted from [10]. Copyright 2012 Nature).

Aromatic regions of the 2D ^13^C–^1^H correlation (HSQC) spectra highlight the differences in the *p*-hydroxyphenyl:guaiacyl:syringyl (H:G:S) distributions in the lignins. Figure 14 shows the significant variations in the compositions of the lignin polymers and other aromatic constituents in the wall. Traditionally, lignin syringyl to guaiacyl ratios (S/G ratios) can be measured by various analytical procedures, but in reality these procedures measure only the S/G of the components released [10]. Although all the methods available, such as thioacidolysis, nitrobenzene oxidation, DFRC, and pyrolysis measures are useful, none of these methods represents the entire lignin. By contrast, the NMR method does represent the entire lignin. By various model studies and by comparisons with other data, where available, the authors are reasonably convinced that NMR-based S/G values are accurate [10]. To obtain these values, only the C–H correlations in similar environments are used, *i.e.*, G_2_ and S_2/6_. Logically, a factor of two is required to adjust for the fact that two symmetrical C–H pairs appear in the contour for syringyl units *versus* one in guaiacyl units. Specially, the S/G ratios could be obtained by the following formula, S/G = IS_2/6_/IG_2_/2. No other corrections are deemed necessary. The NMR profiles of the whole cell walls produced by these methods represent a major advancement in the structural characterization of plant cell wall structures, and they are becoming popular beyond characterizing native cell walls and are now being used for a variety of degraded and industrially pretreated materials. The methods described here are the best currently available, but there remains considerable room for improvement in solvent systems, NMR methods and assignments. Absolute quantification is currently difficult because of the rapid relaxation of the bulk polymer and the much slower relaxation of terminal end units (or pendant units on the polymer, such a *p*-hydroxybenzoates and *p*-coumarates). Thus, volume integrals of these end units over represent their amounts. However, relative quantification is available when comparing the ratio of these components.

**Figure 15:**
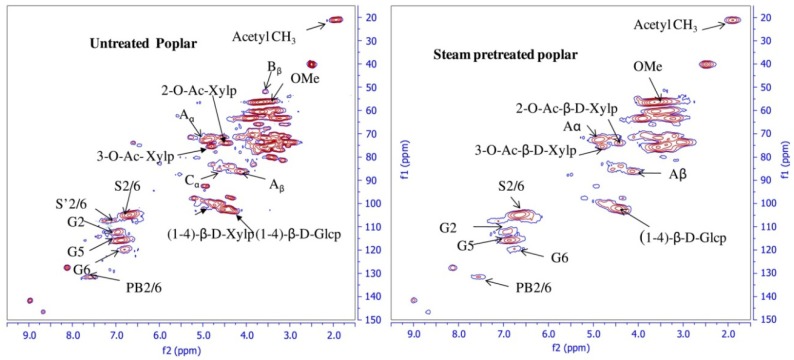

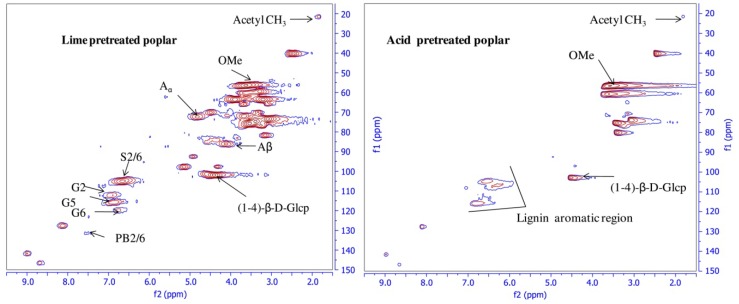
HSQC spectrum of untreated and pretreated poplar in perdeuterated pyridinium chloride-DMSO-*d_6_* system (Reprinted from [82]. Copyright 2011 Elsevier).

The methodology of *in* *situ* characterization of plant cell wall was recently improved by Ragauska’s group [82,83]. They firstly applied *in* *situ* characterization in evaluating the structural changes of biomass pretreatments (steam, dilute H_2_SO_4_ and lime at 160 °C) by developing a novel bi-solvent (per-deuterated pyridinium chloride-DMSO-*d*_6_). All major cell wall components in untreated and pretreated poplar were readily characterized in detail on milligram quantity samples without component isolation [82]. The relative structural changes in lignin subunits (β-aryl ether, resinol, and phenylcoumaran) after pretreatment were estimated from the volume integration of distinguishable cross peaks of various lignin subunits (Figure 15). The degradation of lignin was observed in all pretreatments. 2D-HSQC analysis results were in agreement with the composition analysis of pretreated biomass samples. The methodology was also used to estimate the structural changes of lignin and hemicelluloses of switch grass during various pretreatments [83]. These results seemingly support the structural changes indicated by the spectral data, validating the use of this methodology as a means of characterizing both native and pretreated biomass for the purposes of improving biomass processing and biofuel production technologies.

**Figure 16:**
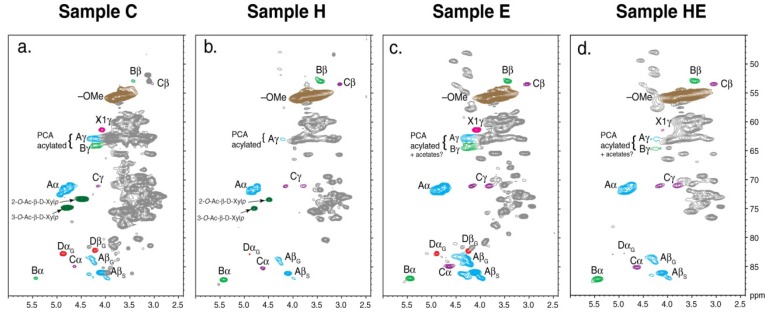

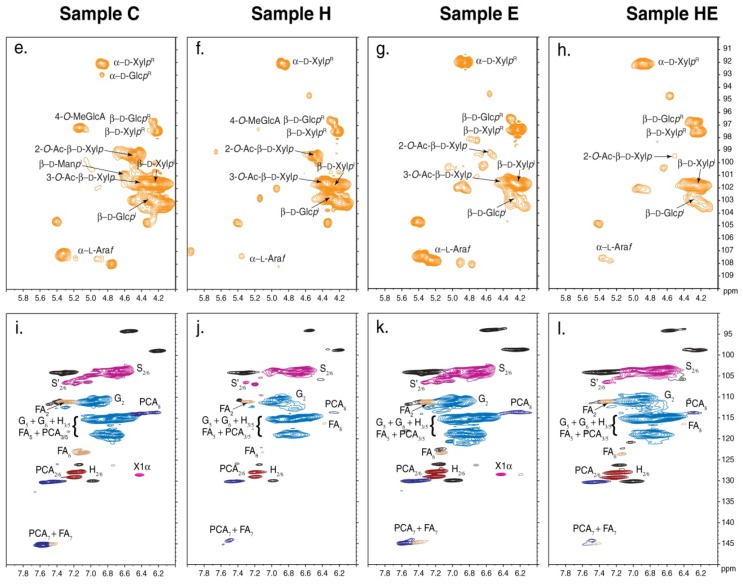
The aliphatic region (**a**–**d**), polysaccharide anomeric region (**e**–**h**), and aromatic region (**i**–**l**) of ^13^C–^1^H correlation (HSQC) spectra of wheat straw. The spectra are aligned vertically to represent each sample treatment as follows: Control (sample C), hydrothermally pretreated (sample H), and enzyme treated (sample E), and hydrothermally pretreated followed by enzyme treated (sample HE). (Reprinted from [69]. Copyright 2013 Springer Link).

Recently, an *in-situ* quantitative 2D-HSQC NMR technique (the ball-milled plant cell wall was dissolved in DMSO-*d*_6_) was used for characterizing the changes in the cell wall during the hydrothermal pretreatment (195 °C for 6 min) process of wheat straw for second-generation bioethanol production [69]. This study provides an effective quantitative method to reveal the structural changes of cell wall components based on *in situ* 2D-HSQC NMR techniques, which was mentioned in Section 2.3.4. The results revealed substantial lignin β-aryl ether cleavage, deacetylation via cleavage of the natural acetates at the 2-*O*- and 3-*O*-positions of xylan, and uronic acid depletion via cleavage of the (1→2)-linked 4-*O*-methyl-α-d-glucuronic acid of xylan after hydrothermal treatment. The aromatic region indicated only minor changes to the aromatic structures during the hydrothermal process (Figure 16). 

## 4. Concluding Remarks

In summary, this paper has reviewed some of the recent literature in the area of lignin characterization (isolated and *in situ* state lignin). The primary focus has been on qualitative and quantitative characterization of lignin by NMR techniques (^13^C-NMR and 2D-HSQC techniques). To achieve optimization of delignification, researchers have devoted their efforts for many years to unveiling the complex chemical structures of lignin polymers. NMR characterization of lignin undoubtedly expands the knowledge of lignin chemistry, which will in return promote the utilization of plant cell walls and high-valued applications of lignin. 

On the other hand, the *in* *situ* 2D-HSQC NMR method has also been used to understand the structural features of lignin in lignocellulosic materials after genetic modifications, chemical, biological processing, and thermo-chemical pretreatments. In this context, solution-state NMR, using modern instruments coupled with modern solution-state NMR pulse experiments, is unparalleled for providing qualitative and quantitative chemical structural features of lignin polymer. From the works reported in this review, it was concluded that novel deuterated solvent systems and high field NMR equipment should be continually developed to characterize lignin structures in an *in situ* state in the future. 

With increased interest in sustainable alternative resources over those from fossil oils for energy and materials production, tremendous efforts are being dedicated to establish processes to transform various biomass feedstocks into liquid fuels and chemicals. As abundant byproducts are collected during various pretreatments and delignification processes, lignin has received considerable attention due to its potential application for industrial utilization. However, value-added utilization of lignin is still hindered by its complex structure and the uncertain reactivity. Therefore, the detailed chemical structures of isolated lignin, which result in different properties of the lignin, should be achieved by advanced NMR techniques, *i.e.*, quantitative ^13^C-NMR and 2D-HSQC techniques. It is believed that the knowledge of structural features of the lignin polymer by NMR techniques will help to maximize the exploitation of lignocelluloses for biorefinery as well as the utilization of isolated lignin for novel materials and chemicals. 
